# Fibroblast growth factor receptor signaling in hereditary and neoplastic disease: biologic and clinical implications

**DOI:** 10.1007/s10555-015-9579-8

**Published:** 2015-07-30

**Authors:** Teresa Helsten, Maria Schwaederle, Razelle Kurzrock

**Affiliations:** Center for Personalized Cancer Therapy and Division of Hematology and Oncology, University of California San Diego, Moores Cancer Center, 3855 Health Sciences Drive, MC #0658, La Jolla, CA 92093-0658 USA

**Keywords:** FGF, FGFR, Cancer, Cancer therapy, Genetics

## Abstract

Fibroblast growth factors (FGFs) and their receptors (FGFRs) are transmembrane growth factor receptors with wide tissue distribution. FGF/FGFR signaling is involved in neoplastic behavior and also development, differentiation, growth, and survival. FGFR germline mutations (activating) can cause skeletal disorders, primarily dwarfism (generally mutations in FGFR3), and craniofacial malformation syndromes (usually mutations in FGFR1 and FGFR2); intriguingly, some of these activating FGFR mutations are also seen in human cancers. FGF/FGFR aberrations reported in cancers are mainly thought to be gain-of-function changes, and several cancers have high frequencies of FGFR alterations, including breast, bladder, or squamous cell carcinomas (lung and head and neck). FGF ligand aberrations (predominantly gene amplifications) are also frequently seen in cancers, in contrast to hereditary syndromes. There are several pharmacologic agents that have been or are being developed for inhibition of FGFR/FGF signaling. These include both highly selective inhibitors as well as multi-kinase inhibitors. Of note, only four agents (ponatinib, pazopanib, regorafenib, and recently lenvatinib) are FDA-approved for use in cancer, although the approval was not based on their activity against FGFR. Perturbations in the FGFR/FGF signaling are present in both inherited and malignant diseases. The development of potent inhibitors targeting FGF/FGFR may provide new tools against disorders caused by FGF/FGFR alterations.

## Introduction

One of the most important advances in tumor biology is the recognition that cancer is frequently driven by inherited or acquired alterations in specific gene(s) or their products [1, 2]. Genomic alterations include changes in expression that can result from mutation, deletion, gene amplification, and/or translocation. Complicating matters, cancers often harbor multiple genetic alterations, but one or a few of these are thought to be primarily responsible for neoplastic behavior in any given tumor. These are the so-called “driver mutations,” while “passenger mutations” may have a more nuanced impact. A literature review suggests that over 1 % of human genes can be implicated as cancer drivers when they are mutated, with protein kinases comprising the largest subgroup of genes altered [3].

Among human signaling pathways, fibroblast growth factor (FGF)/fibroblast growth factor receptor (FGFR) is one of the pathways most enriched in non-synonymous mutations, including several candidate driver mutations [3]. A computational method designed to identify driver mutations within protein kinase datasets successfully identified multiple aberrations in the FGF/FGFR machinery [4]. In keeping with other genes implicated in neoplastic behavior, FGF/FGFR signaling is also involved in development, differentiation, growth, and survival mechanisms. Indeed, FGFR aberrations have been identified in both hereditary and neoplastic human diseases.

Most of the reported FGFR mutations that cause heritable human diseases are activating mutations which increase receptor signaling. These abnormalities are seen in craniofacial and skeletal syndromes such as the craniosynostoses [5–7] (Pfeiffer, Crouzon, Apert, Jackson-Weiss, Muenke, and Beare-Stevenson syndromes) and dwarfism syndromes [8, 9] (achondroplasia, thanatophoric dysplasia, and hypochondroplasia). The only reported inherited condition caused by loss of FGFR function is an autosomal dominant form of hereditary hypogonatotrophic hypogonadism 2 with or without anosmia [10, 11], which is caused by loss of function of FGFR1 [10, 12, 13] or a missense mutation in FGF8 [14]. Intriguingly, some of the same activating FGFR mutations seen in inherited syndromes are also seen in human cancers [14, 15]. Furthermore, FGF/FGFR aberrations reported in cancers are overwhelmingly thought to be gain-of-function changes, including gene amplifications and gene rearrangements [16].

The goal of identification and characterization of driver mutations in cancer is, ultimately, to create successful anti-cancer therapies with which to prosecute these tumors; several such therapies already exist, demonstrating proof of principle [17, 18]. Furthermore, for some gene targets, drugs may impact the course of cancer as well as non-malignant conditions that are driven by abnormalities in the cognate signal. JAK2 aberrations, for instance, are found in myelofibrosis, and JAK2 inhibitors such as ruxolitinib provide significant benefit in such patients [19]. At the same time, the JAK2 inhibitor tofacitinib can benefit patients with rheumatoid arthritis and is approved for that indication [20]. In the case of FGF/FGFR, multiple drugs targeting this pathway have entered the clinic [16]. Herein, we discuss the landscape of diseases that are driven by aberrant FGF/FGFR machinery.

## Molecular biology of FGF/FGFR signaling

Fibroblast growth factors (FGFs) and their receptors (FGFRs) are evolutionarily conserved transmembrane growth factor receptors with wide tissue distribution in all vertebrates. FGFs and FGFRs share homologies among their respective groups and with other signaling molecules. FGFRs in particular are similar to other signaling receptors, including vascular endothelial growth factor receptor (VEGFR), platelet-derived growth factor receptor (PDGFR), and other receptor tyrosine kinases [21]. However, there are important differences between the individual signaling molecules that allow for precise control of a full range of processes, including development, cell survival, differentiation, motility, angiogenesis, and carcinogenesis.

### Receptors

**Fig. 1:**
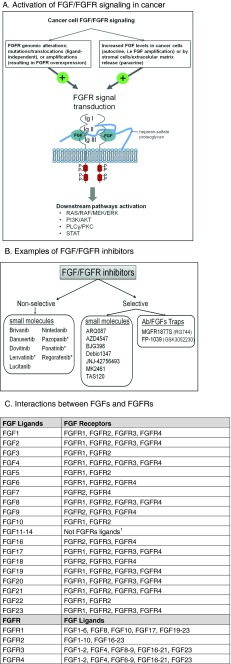
FGF/FGFR activation in cancer and inhibitors. **a** The activation of FGF/FGFR signaling in cancer. The structure of FGF/FGFR comprises two receptor molecules, two FGFs, and one heparan sulfate proteoglycan chain. The FGFRs are formed by three immunoglobulin domains (IgI–III), a transmembrane helix, and intracellular tyrosine kinase domains. The mechanisms driving FGF signaling in cancer can be divided into two categories: first, genomic alterations of FGFR that can lead to ligand-independent receptor signaling and, second, alterations that support a ligand-dependent signaling activation. Following FGF binding to FGFR and heterodimerization, the tyrosine kinase domains phosphorylate each other, leading to the activation of key downstream pathways. **b** Examples of FGF/FGFR inhibitors; *asterisk* denotes FDA-approved drugs in cancer; Ab = Antibody; FP-1039 (GSK3052230) is a ligand trap, i.e., sequesters FGFs and inhibits their signaling [71]. **c** The interactions between FGFs and FGFRs; references: Guillemot et al. [32], Powers et al. [191], Ornitz et al. [192], Zhang et al. [34]. Interaction between FGF ligands and receptors is an evolving field; variability may be observed between studies and tissue types. ^1^FGF11-14 are not ligands for FGFRs and are known as FGF homologous factors (FHF1–4)[28, 29]. There is no human FGF15

In humans, there are five known FGFRs, called FGFR1–FGFR4 and FGFRL1 (also known as FGFR5). FGFR1–FGFR4 are typical growth factor receptor tyrosine kinases, with extracellular immunoglobulin (Ig)-like domains and intracellular tyrosine kinase domains, while FGFRL1 lacks the intracellular kinase domain and has less clear function(s)[22, 23]. Upon binding of their ligands, the typical receptors homo- or hetero-dimerize, leading to sequential phosphorylation of specific intracellular tyrosine residues and activation of an intracellular signaling cascade and gene transcription [24] (Fig. 1). The FGFR signaling pathway interacts with several other important intracellular pathways, including PI3K/Akt, Wnt, hedgehog, and bone morphogenic protein (BMP) [24, 25]. FGFR1–4 have different ligand specificities based on developmental aspects, tissue distribution, and RNA splicing variation [26]. For example, the FGFR2b isoform is predominantly expressed in epithelial cells, while the FGFR2c isoform is expressed predominantly in mesenchymal cells, and switching from FGFR2b to FGFR2c occurs during progression and invasion of prostate and bladder cancers [27].

### Ligands

There are 18 human ligands for FGFRs (Fig. 1). They are FGF1 (acidic FGF), FGF2 (basic FGF), FGF3, FGF4, FGF5, FGF6, FGF7 (KGF), FGF8, FGF9, FGF10, FGF16, FGF17, FGF18, FGF19, FGF20, FGF21, FGF22, and FGF23. FGF11–14 are not ligands for FGFRs and are known as FGF homologous factors (FHF1–4) [28, 29]. There is no human FGF15 (FGF15 is the mouse equivalent of human FGF19) [30]. The FGFR ligands are secreted proteins that bind to the extracellular matrix, thereby restricting their influence to the tissue where they are produced (autocrine or paracrine function). However, three of the FGFs—FGF19, FGF21, and FGF23—bind less tightly to extracellular matrix heparin sulfates, so they are able to act systemically in an endocrine fashion (hormone-like), allowing them to spread from their production site into the circulation [30, 31]. Most FGFs are secreted proteins with cleavable amino terminal portions, but FGF9, FGF16, and FGF20 have non-cleavable secretion sequences, and FGF1 and FGF2 have no secretion sequences, although they are found in the extracellular compartment, suggesting an alternate process of release [29, 32]. Most FGFs are released from binding to the extracellular matrix via action of heparin sulfate proteoglycans and/or fibroblast growth factor–binding protein (FGFBP1)[33].

### FGF/FGFR interaction and function

In general, FGFs are promiscuous, and most can bind to any of the four main FGFRs, although some of the FGF/FGFR ligand/receptor pairs differ in their affinities (Fig. 1c). FGFs interact with cell surface heparan sulfate proteoglycans (HSPGs) and with the transmembrane protein Klotho (for the hormone-like FGF19, 20, 23) to stabilize binding to FGFRs. Ornitz et al. [34, 35] systemically investigated affinities of FGFs for each FGFR isoform and showed, for example, that FGFR2b is a high-affinity receptor for FGF1, FGF7, FGF10, and FGF22, while FGFR2c has high affinity for FGF1, FGF2, FGF4, FGF6, FGF8, FGF9, and FGF20. But, differential expression of either FGFs or FGFRs in time or tissue may also contribute to tissue-specific effects of FGF/FGFR signaling. For example, FGF1 and FGF2 are expressed in both embryonic and adult tissues, while FGF7–FGF9 are predominantly expressed in developing or in restricted adult tissues [36].

The differences in activity/function of the different FGF/FGFR pairs are also highlighted by murine gene knockout experiments. Mice heterozygous for FGFR knockout mutations develop normally, so haplo-insufficiency is not likely to be a factor [37]. However, mice homozygous for FGFR1 or FGFR2 null mutations die *in utero*, and FGFR3-null mice develop normally other than overgrowth of cancellous bones and deafness [37]. Most of the FGFs have also been knocked out in mouse models, with varying phenotypic effects, including lethality (FGF4, FGF8, FGF9, FGF10, FGF19, FGF18, and FGF23), defects in neuromusculoskeletal development or function (FGF2, FGF3, FGF6, FGF7, FGF12, FGF14, FGF17, FGF18), cardiac defects (FGF2, FGF9, FGF16, FGF19), and no identifiable abnormality (FGF1) [29].

### Activation of signaling

To signal, FGFs have to be released from the extracellular matrix by heparinases, proteases, or specific fibroblast growth factor–binding protein (FGFBP1). FGFBP1 are secreted heparin proteins that reversibly bind FGF1 and FGF2, releasing them from the extracellular matrix and increasing the local levels of free ligand available for receptor binding [38]. FGFBP1 is secreted by keratinocytes and human epidermal carcinomas, particularly squamous cell carcinomas [33, 38]. Its expression is seen in development, wound healing, cancer, and angiogenesis, and it is downregulated by pharmacologic agents *in vitro* [33]. The fibronectin-leucine-rich transmembrane protein 3 (FLRT3) is co-expressed with FGF8 during development, co-immunoprecipitates with FGF8/FGFRs, physically interacts with FGFRs via its fibronectin type III domain, and increases activity through the MAPK pathway, facilitating FGFR signaling [39, 40].

### Inhibition of signaling

FGF/FGFR signaling is negatively regulated by several mechanisms. FGFR stimulation activates sprouty proteins (SPRY1–4), which in turn negatively feedbacks on FGF/FGFR/MAPK signaling by interacting with growth factor receptor bound protein 2 (GRB2), son of sevenless homolog 1 (SoS1), and/or RAF proto-oncogene serine/threonine-protein kinase (RAF1) [41, 42]. Intriguingly, sprouty proteins are thought to be general inhibitors of receptor tyrosine kinase growth factor signaling via inhibition of Ras-MAPK signaling, but their behavior differs among specific growth factor receptors. For example, sprouty protein inhibits FGFR signaling but potentiates epidermal growth factor receptor (EGFR) signaling [43]. The cytoplasmic domain of similar expression to FGF (SEF, also known as IL-17 receptor D) interacts with the cytoplasmic domain of FGFR and inhibits downstream signaling of the pathway [43]. Finally, MAP kinase phosphatases (MKPs) dephosphorylate ERKs and thereby downregulate the signaling pathway. MKP3 functions within the cytoplasm, whereas MKP1 is localized in the nucleus [43].

FGFRL1 is the atypical receptor that lacks an intracellular kinase domain. Like the other FGFRs, it is found in all vertebrates, is expressed in a wide variety of tissues, and binds to some FGF ligands. *In vitro* experiments show that FGFRL1 binds FGF3, FGF4, FGF8, FGF10, and FGF22, but not the other FGFs tested [44]. Unlike FGFR1–4, it is shed from cell membranes [44], suggesting that it may serve as a ligand trap to negatively regulate signaling [45]. Other regulatory mechanisms might include heterodimerization with other FGFRs to prevent trans-autophophorylation and thereby negatively regulating intracellular signaling or by increasing membrane turnover rates of the other FGFRs, but there is currently no evidence that these mechanisms exist [45]. The exact functions of FGFRL1 are not known. FGFRL1-null mice die in infancy with diaphragmatic defects and renal agenesis [45], and there are case reports of FGFRL1 mutations in human disease: craniosynostosis [46] and ovarian cancer [47].

## FGF/FGFR aberrant signaling in human disease

### Inherited syndromes

Several types of inherited syndromes are due to germline aberrations in FGFR. These include craniosynostosis syndromes and achondroplasia [48], mainly due to gain-of-function mutations, as well as loss-of-function anomalies associated with congenital hypogonadotropic hypogonadism (Table 1). Interestingly, although some of these aberrations are identical to those that, in somatic form, are associated with cancer, for the most part, the individuals with these hereditary syndromes have not been reported to have an increased incidence of cancer. Apart from case reports [49–53], there is no epidemiologic evidence that people with craniosynostosis, dwarfism, or congenital hypogonadotropic hypogonadism are at increased risk for malignancy. This is possibly related to differential effects of activating FGFR mutations in cell or tissue type in the context of stage of development. It is possible, for instance, that there are as yet unknown anti-neoplastic compensatory effects in individuals with these disorders, perhaps stemming from the presence of germline FGFR aberrations during development as opposed to the appearance of somatic FGFR mutations in human cancers.

**Table 1:** Examples of FGFR aberrations in inherited syndromes

Gene chromosome aberration	Syndrome (OMIM number)	Clinical features	Examples of cancers in which an aberration is seen	Reference(s)
FGFR1 (Chr 8p)				
P252R	Pfeiffer syndrome, type I (01600)	*Type 1* “classic” Pfeiffer syndrome: mild manifestations, ibrachycephaly, midface hypoplasia, finger and toe abnormalities, normal intelligence, and generally good outcome	None reported	[83–86] [87, 88]
Y372C	Osteoglophonic dysplasia (166250)	Craniosynostosis, telechanthus, facial hypoplasia, prominent supraorbital ridge, depressed nasal bridge, and rhizomelic dwarfism	None reported	[83, 89, 90]
FGFR2 (Chr 10q) S252W or P253R (most common)	Apert syndrome (101200)	Craniosynostosis, midface hypoplasia, syndactyly of the hands and feet, tendency to fusion of bony structures, varying mental deficiency, and hearing loss. Increased number and maturation of pre-osteoblasts	Endometrial cancers (S252W and P253R)	[7, 83, 91, 92] [93, 94]
Multiple mutations reported [95]	Crouzon syndrome (123500)	Craniosynostosis, hypertelorism, exophthalmos, external strabismus, parrot-beaked nose, short upper lip, hypoplastic maxilla, and relative mandibular prognathism	Gastric cancer (S267P)	[83, 95–97] [98]
S252L, S267P	Pfeiffer syndrome, type 2 and 3 (101600)	*Type 2*: cloverleaf skull with “Pfeiffer” hands and feet, ankylosis of the elbows. *Type 3*: similar to Type 2, but without cloverleaf skull. Early demise is characteristic of types 2 and 3	Gastric cancer (S267P)	[84, 99, 100] [98]
FGFR3 (Chr 4p)				
G380R, S279C G375C	Achondroplasia (100800)	Most frequent form of dwarfism: short stature, rhizomelic shortening of limbs, frontal bossing, midface hypoplasia, exaggerated lumbar lordosis, limitation of elbow extension, genu varum, and trident hand	Bladder, prostate, and testicular cancers (G380R)	[8, 83, 101–103] [104, 105]
R248C, S249C, R373C, Ter807G/R/C, G370C, N540L, Q485R	Thanatophoric dysplasia I (TDI) (187600)	Severe dwarfism; usually fatal in the neonatal period. Curved short femurs with or without cloverleaf skull	Bladder (R248C, S249C, G370C), Prostate (S249C), Lung squamous (R248C, S249C), Head and Neck (S249C), Multiple Myeloma (R248C)	[83, 106–108] [109–111]
K650E	Thanatophoric dysplasia II (187601)	Severe dwarfism; usually fatal in the neonatal period. Straight, short femurs with cloverleaf skull	Multiple Myeloma, Bladder, Glioblastoma	[83, 112, 113] [56, 114]
N540K/T/S I538V, K650N/Q, L652Q, Y278C S84L	Hypochondroplasia (146000)	Dwarfism, lumbar lordosis, short and broad bones, and caudal narrowing of the interpediculate distance of the lumbar spine. Some resemblance to achondroplasia, but is much milder	Renal cell carcinoma (K650N)	[83, 115–118] [56]
P250R	Muenke syndrome (602849)	Coronal synostosis, macrocephaly, midface hypoplasia, developmental delay. Variable phenotype	None reported	[5, 83, 86, 119]

*Chr* chromosome, *FGF* fibroblast growth factor, *FGFR* fibroblast growth factor receptor, *OMIM* Online Medelian Inheritance in Man (http://www.omim.org/)

### Somatic aberrations in benign conditions

Somatic or acquired mutations in FGFR3 have been observed in benign skin conditions like seborrheic keratosis and epidermal nevi [54, 55]. These aberrations are activating and, when evaluated, are not seen in adjacent normal skin [55]. Many of these are identical to mutations that are also seen in bladder and/or cervical cancers.

### Somatic aberrations in malignancies

**Fig. 2:**
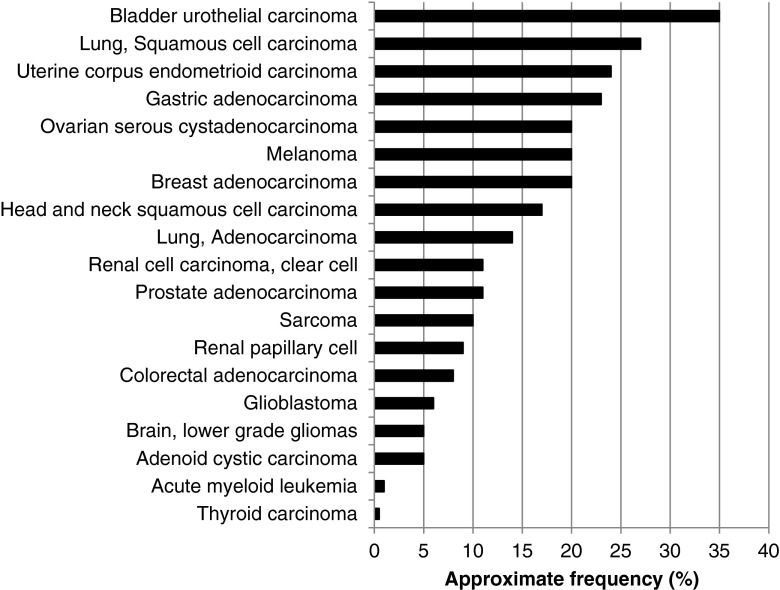
Approximate frequencies of FGFR alterations in diverse cancers. Data was extracted/analyzed based on cbioportal at http://www.cbioportal.org/public-portal (accessed November 2014). Most of the studies included >200 patients. Alterations in FGFR1, FGFR2, FGFR3, and FGFR4 were included. Please refer to Table 3 for more details and additional references. Abbreviations: *FGFR* = fibroblast growth factor receptor

**Fig. 3:**
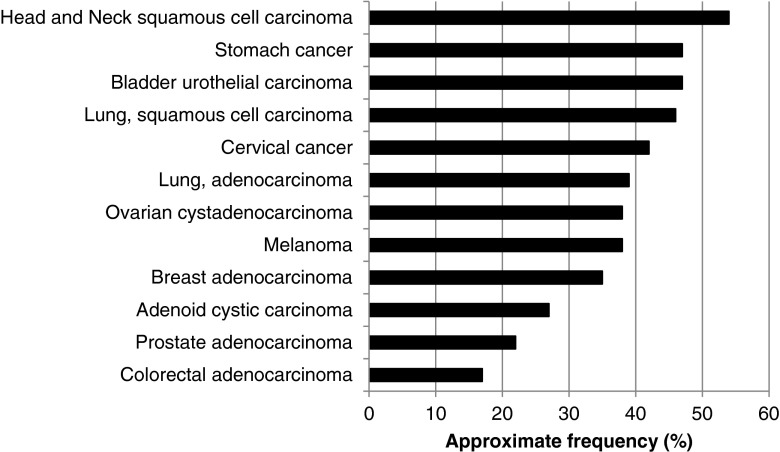
Approximate frequencies of FGF ligand alterations in diverse cancers. Data was extracted/analyzed based on data from *cbioportal* at http://www.cbioportal.org/public-portal (accessed November 2014). Cervical cancer included squamous cell carcinoma and endocervical adenocarcinoma. Please refer to Table 4 for more details and additional references. Abbreviations: *FGF* = fibroblast growth factor

Aberrations in FGFR and its ligands are common in malignancy (Tables 2, 3, and 4 and Figs. 2 and 3). Across malignancies, FGF anomalies are found in ≈14 % and FGFR in ≈7 % of malignancies [56] (FGF/FGFR in about 20 %).

**Table 2:** Specific examples of FGFR alterations in cancer

Activating aberrations	Examples of disease(s) (most common)	Reference(s)
Amplifications		
FGFR1	Squamous cell carcinoma of lung, breast adenocarcinoma, bladder urothelial carcinoma, head and neck squamous cell carcinoma	[78, 120–123] [124] [125, 126]
FGFR2	Gastric adenocarcinoma	[127–129]
FGFR3	Uterine carcinosarcoma, ovarian cystadenocarcinoma, sarcoma	[56] [130] [56, 131]
FGFR4	Kidney, renal clear cell carcinoma	[56]
Mutations		
FGFR1	Stomach adenocarcinoma, melanoma	[127] [56, 132]
FGFR2	Uterine (endometrial carcinoma), melanoma^a^	[133] [56, 59, 132]
FGFR3	High-grade bladder cancer, cervical cancer	[124, 134, 135] [136]
FGFR4	Rhabdomyosarcoma, melanoma	[137, 138] [132]
Rearrangements		
FGFR1	8p11 myeloproliferative syndrome/fusions partners: *BCR*, *ZNF198*, *CEP110*, *FGFR1OP1*, *FGFR1OP2*, *HERVK*, *TRIM24*, *LRRFIP1*, *MYO18A*, *CPSF6*	[63]
	Rhabdomyosarcoma/fusions partner: *FOXO1*	[64]
	Glioblastoma/fusions partner: *TACC1*	[68]
	Salivary gland/fusions partner: *PLAG1*	[139]
FGFR2	Cholangiocarcinoma/fusions partners: *AHCYL1,BICC1*	[140, 141]
	Breast/fusions partners: *AFF3, CCDC6, CASP7*	[141]
FGFR3	Multiple myeloma/fusions partner: *MMSET*	[65]
	Glioblastoma, bladder carcinoma, head and neck squamous cell carcinoma/fusions partner: *TACC3*	[68, 135, 141]

^a^FGFR2 mutations may be loss of function

**Table 3:** Examples of FGFR mutations and copy number alterations in cancer

Type of cancer	Approximate frequency	Approximate frequencies by FGF receptor^a^	Comments	References
Bladder urothelial carcinoma	35 %	FGFR1 14 %FGFR3 19 % FGFR2 3 %FGFR4 6 %	FGFR1 almost all amplifications FGFR3 mostly amplification and mutations	[56, 124]
Lung, squamous cell carcinoma	27 %	FGFR1 18 %FGFR3 4 % FGFR2 4 %FGFR4 2 %	FGFR1: Almost all are gene amplifications	[56, 142]
Uterine corpus endometrioid carcinoma	24 %	FGFR1 7 %FGFR3 5 % FGFR2 14 %FGFR4 4 %	FGFR1 approximately 50 % amplification and 50 % mutations FGFR2 almost all mutations	[56, 133]
Gastric adenocarcinoma	23 %	FGFR1 6 %FGFR3 4 % FGFR2 10 %FGFR4 5 %	Approximately 50 % amplifications/deletions and 50 % mutations	[56, 127]
Breast adenocarcinoma	20 %	FGFR1 14 % FGFR3 2 % FGFR2 3 %FGFR4 2 %	Almost all are amplifications	[56, 120, 143–145]
Melanoma	20 %	FGFR1 5 %FGFR3 5 % FGFR2 11 %FGFR4 5 %	FGFR2 mostly mutations	[56]
Ovarian serous cystadenocarcinoma	20 %	FGFR1 5 %FGFR3 8 % FGFR2 4 %FGFR4 4 %	Almost all amplifications, rare mutations	[56, 130]
Head and neck squamous cell carcinoma	17 %	FGFR1 10 %FGFR3 4 % FGFR2 1 %FGFR4 1 %	Majority of amplification with about 20 % deletion and mutations (each), and few fusions	[56, 146, 147]
Lung, adenocarcinoma	14 %	FGFR1 6 %FGFR3 2 % FGFR2 4 %FGFR4 4 %	Approximately 50 % amplifications and 50 % mutations, with predominance of FGFR1 amplification	[56, 148–150]
Prostate adenocarcinoma	11 %	FGFR1 6 %FGFR3 1 % FGFR2 3 %FGFR4 1 %	Approximately 50 % amplification, 50 % deletions, mutations rare	[56, 151]
Renal cell carcinoma, clear cell	11 %	FGFR1 2 %FGFR3 1 % FGFR2 < 1 %FGFR4 7 %	Majority amplifications	[56, 152]
Sarcoma	10 %	FGFR1 4 %FGFR3 4 % FGFR2 1 %FGFR4 2 %	Majority amplifications (*n* = 2 deletions)	[56, 131]
Renal papillary cell	9 %	FGFR1 4 %FGFR3 2 % FGFR2 1 %FGFR4 3 %	All mutations, only 2 cases had amplification	[56]
Colorectal adenocarcinoma	8 %	FGFR1 5 %FGFR3 1 % FGFR2 1 %FGFR4 1 %	FGFR1 about 60 % amplification, rest mutations/deletion	[56, 153]
Glioblastoma	6 %	FGFR1 0 %FGFR3 2 % FGFR2 3 %FGFR4 1 %	FGFR2 mostly deletions	[56, 154]
Adenoid cystic carcinoma	5 %	FGFR1 3 %FGFR3 0 % FGFR2 0 %FGFR4 2 %	FGFR1 amplification and deletion (1 each) FGFR4 mutation (*n* = 1)	
				[56, 155]
Brain, lower grade gliomas	5 %	FGFR1 0 %FGFR3 1 % FGFR2 3 %FGFR4 1 %	Most are deletions, with few amplifications and mutations	[56, 156]
Acute myeloid leukemia	1 %	FGFR1 < 1 %FGFR3 0 % FGFR2 0 %FGFR4 < 1 %	1 amplification, 1 deletion, no mutations	[56, 157]
Thyroid carcinoma	<1 %	FGFR1 0 %FGFR3 < 1 % FGFR2 < 1 %FGFR4 < 1 %	Two amplifications, one mutation	[56]

See also Fig. 2 for illustration (bar graph)

*FGF* fibroblast growth factor, *FGFR* fibroblast growth factor receptor

^a^Data extracted/analyzed based on cbioportal at http://www.cbioportal.org/public-portal (accessed November 2014). Most of the studies included >200 patients

**Table 4:** Examples of FGF ligand mutations and copy number aberrations in cancer

Type of cancer	Approximate frequency	Approximate frequencies by FGF ligand^a^	Comments	Reference(s)
Head and neck squamous cell carcinoma	54 %	FGF3 28 %FGF12 19 % FGF4 28 %FGF10 6 % FGF19 28 %FGF23 5 %	Virtually all amplifications	[56]
Bladder urothelial carcinoma	47 %	FGF3 13 %FGF1711% FGF4 12 %FGF10 9 % FGF19 13 %FGF20 9 %	FGF3, FGF4, and FGF19 co-amplified in approximately 12 % of cases FGF17 and 20 mostly deletions	[56, 124]
Stomach cancer	47 %	FGF3 7 %FGF12 8 % FGF4 7 %FGF13 6 % FGF19 7 %FGF14 5 % FGF10 9 %FGF17 5 %	FGF3/4/19 co-amplified in 7 % of cases FGF17 and FGF20 both deleted in 2 % of cases	[56, 127]
Lung, squamous cell carcinoma	46 %	FGF3 12 %FGF12 26 % FGF4 12 %FGF10 7 % FGF19 13 %	Virtually all are gene amplifications	[56, 142]
Cervical cancer^b^	42 %	FGF12 25 %	All are amplifications	[56]
Lung, adenocarcinoma	39 %	FGF10 11 % FGF17 7 % FGF20 7 %	FGF10 mostly amplifications FGF17 and FGF20 mostly deletions FGF3/4/19 co-amplified in 4 % of cases	[56, 148–150]
Melanoma	38 %	FGF3 8 % FGF4 6 % FGF19 6 %	FGF3/4/19 co-amplified in about 7 % of cases	[56]
Ovarian cystadenocarcinoma	38 %	FGF3 5 %FGF6 5 % FGF4 4 %FGF23 6 % FGF19 4 %FGF12 13 %	Virtually all amplifications	[56, 130]
Breast adenocarcinoma	35 %	FGF3 15 %FGF17 6 % FGF4 15 %FGF20 5 % FGF19 15 %	High frequency of co-amplification of FGF3/4/19. Similar results with TCGA, Nature 2012 study (*n* = 482)	[56, 120, 143, 145]
Adenoid cystic carcinoma	27 %	FGF22 10 % All others 5 %or less	Approximately 50 % deletions and 50 % amplifications, rare mutations	[56, 155]
Prostate adenocarcinoma	22 %	FGF17 8 % FGF20 5 %	Majority are deletions, about 5 % cases are co-deleted FGF17/20	[56, 151]
Colorectal adenocarcinoma	17 %	All 5 % or less	Majority of mutations, less amplifications and rare deletion	[56, 153, 158]

See also Fig. 3 for illustration (bar graph)

*FGF* fibroblast growth factor

^a^Included FGFs with alteration frequency ≥5 % and at least 5 cases with the alteration. Extracted/analyzed in part based on cbioportal at http://www.cbioportal.org/public-portal (accessed November 2014)

^b^Squamous cell carcinoma and endocervical adenocarcinoma. Abbreviations: FGF = fibroblast growth factor. See also Fig. 3 for illustration (bar graph)

#### FGFR alterations

While almost any FGFR genes can be altered in many cancer types, some acquired genetic aberrations are more striking in prevalence or in cancer type(s) than others. The most common abnormalities reported to date are gene amplifications of FGFR1–3 (Table 2). These are generally assumed to represent activation or gain-of-function amplifications, but full molecular characterization of the effects of gene amplification within specific cellular or cancer microenvironment contexts is not fully available. The cancers in which FGFR gene amplifications are most frequent include bladder urothelial carcinomas (FGFR1), squamous cell lung cancer (FGFR1), head and neck squamous cell cancer (FGFR1), uterine carcinosarcoma (FGFR3), breast adenocarcinoma (FGFR1), and gastric adenocarcinoma (FGFR2) (Tables 2 and 3, and Fig. 2). In fact, FGFR2 was first identified in a gastric cancer cell line, and it is preferentially amplified/overexpressed in the diffuse type of gastric cancer, which correlates with poor prognosis, at least in a Japanese population [57]. More commonly, however, mutations in FGFR3 characterize bladder carcinoma. Indeed, activating mutations of fibroblast growth factor receptor-3 (*FGFR3*) have been described in approximately 75 % of low-grade papillary bladder tumors. In muscle-invasive disease, *FGFR3* mutations are found in 20 % of tumors, but overexpression of FGFR3 is observed in about half of cases [58].

Loss-of-function alterations are relatively uncommon in cancer (a pattern that is also seen in hereditary disorders). However, there is at least one report indicating that some melanoma cell lines demonstrate FGFR2 loss of function [59].

FGFR1 amplification is prevalent in both squamous cell carcinoma of the lung and of the head and neck, suggesting a possible common underlying mechanism of carcinogenesis in these smoking-related carcinomas. Perhaps more importantly, development of FGFR1 inhibitors represents a viable targeted therapy for use in squamous cell lung cancers [60, 61].

#### FGF alterations

FGF abnormalities are for the most part amplifications (Fig. 3 and Table 4). There are few reported data regarding the mechanism(s) of action of these amplifications. Nevertheless, it seems reasonable to hypothesize that abundance of ligand could lead to increased receptor signaling, but the reality may be more complex, perhaps relating to the stoichiometric proportions of the different ligands present. Of note, three of the ligands, FGF3, FGF4, and FGF19, are frequently co-amplified on 11q13 [62]. This amplicon is present in several cancers, including breast, bladder, and squamous cell carcinoma of the lung and head and neck (Fig. 3 and Table 4).

#### FGFR rearrangements

Chromosomal translocation gives rise to chimeric gene products with aberrant function (Table 2). In general, fusion gene proteins result from the fusion of a “partner gene” with a tyrosine kinase domain derived from the *FGFR* family member gene. As a result of their constant dimerization state, they are constitutively active in the absence of ligand. The *FGFR1* gene can be fused to other genes including *BCR*, *ZNF198*, *CEP110*, *FGFR1OP1*, *FGFR1OP2*, *HERVK*, *TRIM24*, *LRRFIP1*, and *MYO18A*, in the 8p11 myeloproliferative syndrome manifested by myeloproliferative neoplasms and peripheral blood eosinophilia without basophilia [63]; it is fused to the *FOXO1* gene in alveolar rhabdomyosarcoma, and the *FOXO1-FGFR1* fusion gene is amplified [64]. The *FGFR3* gene is fused to the *MMSET* gene as a result of a t(4;14)(p16.3;q32) chromosomal translocation in 10–20 % of multiple myeloma [65]; it is fused to the *ETV6* gene in peripheral T cell lymphoma with a t(4;12)(p16;p13) chromosomal translocation [66]. In glioblastoma multiforme, *FGFR1* and *FGFR3* genes are fused to neighboring *TACC1* and *TACC3* genes due to interstitial deletions, respectively [67, 68].

## FGF/FGFR signaling inhibitors and cancer therapy

### FDA approved drugs that target FGFR

Only four drugs (ponatinib, regorafenib, pazopanib, and more recently lenvatinib) are FDA-approved for use in human cancers (Table 5). Ponatinib is a multi-tyrosine kinase inhibitor that was approved for imatinib-resistant chronic myelogenous leukemia (CML) and Philadelphia chromosome-positive (CP) acute lymphoblastic leukemia (ALL). The efficacy results demonstrated a 54 % major cytogenetic response (MCyR) rate in patients with CP-CML, and seventy percent of patients with CP-CML with the T315I mutation in BCR-ABL achieved MCyR. In addition of targeting BCR-ABL, ponatinib can also inhibit members of the VEGFR, PDGFR, FGFR (IC_50_ = 2 nM for FGFR1), and SRC families of kinases, KIT, or RET, with IC_50_ between 0.1 and 20 nM. Of note, ponatinib was briefly taken off the market by the FDA because of concerns about cardiovascular side effects (clotting), but soon after, it was returned to the market with updated safety monitoring recommendations. Regorafenib was approved for imatinib-resistant gastrointestinal stromal tumor (GIST) and metastatic colorectal cancer, based on a statistically significant survival prolongation observed in patients randomized to receive regorafenib (6.4 vs 5.0 months in the placebo arm, *P* = 0.01). Regorafenib, and its active metabolites inhibit multiple membrane-bound and intracellular kinases including those in the RET, VEGFR1, VEGFR2, VEGFR3, KIT, PDGFR-α and -β, FGFR1-2, and Abl pathways. Pazopanib was approved for advanced renal cell carcinoma (based on a progression-free survival of 9.2 months compared to 4.2 months in the placebo arm), as well as soft tissue sarcomas (improved progression-free survival: 4.6 months versus 1.6 for patients who received placebo). Pazopanib is a multi-tyrosine kinase inhibitor whose targets include VEGFR1-3, PDGFR-α and -β, FGFR-1 and -3, and KIT. Lastly, lenvatinib is a multi-kinase inhibitor (targets including VEGFR1–3, FGFR1–4, PDGFR-α, KIT, and RET) indicated for the treatment of patients with locally recurrent or metastatic, progressive, radioactive iodine-refractory differentiated thyroid cancer. The recent approval in February 2015 was based on an improved progression-free survival (18.3 vs 3.6 months in the placebo group, *P* < 0.001). The approvals of these four agents (ponatinib, regorafenib, pazopanib, and lenvatinib), all three of which are multi-kinase inhibitors, were not based on activity against FGFR. Of interest, nintedanib is an FGFR inhibitor that is FDA-approved for a non-cancer indication—idiopathic pulmonary fibrosis (Table 5).

**Table 5:** Examples of drugs that inhibit FGF/FGFR pathway signaling

Drug	Company/type of drug	Examples of target(s)	FDA-approved Yes/No	Examples of clinical development/trials/phase	References
ARQ087	ArQule/ selective FGFRs inhibitor	FGFR1/2/3	No	Phase I Dose Escalation Study	[159]
AZD4547	Astrazeneca/ selective FGFRs inhibitor	FGFR1/2/3	No	Phase II Part of Lung-MAP	[128, 160, 161]
Brivanib (BMS-540215)	BMS/ dual kinase inhibitor	FGFR1/2/3, VEGFR	No	Phase III hepatocellular; did not meet endpoint of survival non-inferiority	[121, 162–164]
Danusertib (PHA-739358)	Nerviano Medical Sciences/multi-kinase inhibitor	FGFR1, BCR-Abl, c- RET, Aurora	No	Phase II in unselected prostate cancer showed minimal activity	[165, 166]
Debio1347	Debiopharm/ selective FGFRs inhibitors	FGFR1/2/3	No	Phase I (selecting patient with FGFRs alterations)	[70, 159]
Dovitinib (TKI 258)	Novartis/ multi-kinase inhibitor	FGFR1/3, PDGFR, VEGFR, Flt3, c-kit	No	Phase III: renal cell carcinoma (failed to meet the primary endpoint, unselected patients)	[167–169]
FP-1039 (GSK3052230)	GlaxoSmithKline/ FGFsFGFs trap agent	Sequesters	No	Phase I (selecting patients with deregulated Fibroblast Growth Factor (FGF) Pathway Signaling)	[71]
JNJ-42756493	Janssen/ selective FGFRs inhibitors	FGFR1/2/3/4	No	Phase I	[159, 170]
Lenvatinib (E7080)	Esai/ multi-kinase inhibitor	PDGFR	Yes	Phase III:FDA- approved for thyroid cancer (Feb 2015)	[171, 172]
Lucitanib (E3810)	Clovis/ dual kinase inhibitor	FGFR1/2 and VEGFR1/2/3	No	Phase II: ER-positive breast cancer	[69, 173–175]
MGFR1877S (RG744)	Genentech/ monoclonal antibody	Anti-FGFR3	No	Phase I	[176]
MK2461	Merck/ multi-kinase inhibitor	FGFR1/2/3, PDGFR, c-Met, Flt1/3, Ron, Mer	No	Phase II	[177]
Nintedanib (BIBF1120)	Boehringer Ingelheim/ multi-kinase inhibitor	FGFR1/2/3, VEGFR, PDGFR, flt3	Yes (not in cancer)	Phase III: ovarian, lung, FDA-approved for idiopathic pulmonary fibrosis (Oct 2014)	[178–180]
NVP-BGJ398	Novartis/ selective FGFRs inhibitor	FGFR1/2/3	No	Phase II	[181–183]
Pazopanib	GlaxoSmithKline/ multi-kinase inhibitor	FGFR1/3, VEGFR1/2/3, PDGFR, c-Kit	Yes	Approved for advanced renal cell carcinoma and soft tissue sarcoma	[184, 185]
Ponatinib	Ariad/ multi-kinase inhibitor	FGFR1-4, BCR-Abl, PDGFR, VEGFR	Yes	Approved for T315I-positive chronic myelogenous leukemia and Ph-positive acute lymphoblastic leukemia	[186, 187]
Regorafenib	Bayer/ multi-kinase inhibitor	FGFR1/2, RET, VEGFR1/2/3 KIT, PDGFRs	Yes	Approved for advanced GIST and colorectal cancer (no FGFR selection)	[188, 189]
TAS120	Taiho Oncology/ selective FGFRs inhibitors	FGFR1/2/3/4	No	Phase I/II (selecting patient with FGFRs alterations)	[159, 190]

The IC_50_ (half-maximal inhibitory concentration) was <100 nmol/L for all the drugs included, except regorafenib

*FGFR* fibroblast growth factor receptor, *GIST* gastrointestinal stromal tumor

### Experimental agents that target FGF/FGFR

There are several pharmacologic agents that have been or are being developed for inhibition of FGFR via targeting of the ATP binding site of the intracellular tyrosine kinase domain(s) (Table 5). The inhibition varies by their affinities for FGFR signaling, as most of these molecules are promiscuous kinase inhibitors (inhibiting VEGF, PDGF, and many other TKIs in addition to FGFR). Figure 1b gives examples of selective versus non-selective FGFR inhibitors. Of note, the dual kinase inhibitor (VEGFR/FGFR) lucitanib has shown activity in FGFR1-amplified breast cancer, with an overall response rate of 50 % [69]. There are also selective tyrosine kinase inhibitors (TKIs) available (Table 5 and Fig. 1b). While several of these agents are currently in clinical trials, none of the more highly selective FGFR inhibitors (e.g., BGJ398 or AZD4547) are currently FDA-approved. A recent study demonstrated that Debio 1347 (a selective orally available FGFR1–3 inhibitor) displayed preferential anti-tumor activity against cells with FGFR genetic alterations in a panel of 327 cancer cell lines and xenograft models [70]. Debio 1347 is currently under investigation for the treatment of patients harboring FGFR genetic alterations.

Of interest, other types of agents have been developed. As an example, FP-1039 is a soluble fusion protein, consisting of the extracellular domains of human FGFR1 linked to the Fc region of the human immunoglobulin G1; it is designed to bind multiple FGF ligands [71] (TRAP molecule).

A large number of other drugs and indications are being pursued. Some FGFR inhibitors have failed to meet their phase III endpoints (Table 5). The majority of the failed trials, however, have been performed in patient populations that were not biomarker-selected.

### Resistance mechanisms

In a recent phase 1 study reporting on patients with FGFR1-amplified (identified by fluorescent/chromogenic *in situ* hybridization) advanced or metastatic lung squamous cell carcinoma (SCC) treated with the selective pan-FGFR inhibitor BGJ398, only a limited number of patients achieved relatively short-lived partial responses (2 of 17 patients, 11.7 %); responses lasted 3 and 8 months. These observations suggest the existence of resistance mechanisms. Sohl et al. [72] reported that resistance mutations at the “gatekeeper” residue may arise (FGFR1 V561M mutation confers a 38-fold increase in autophosphorylation and significant resistance to lucitanib), leading to tumor progression and explaining the non-durable responses. For instance, it has also been shown that the heterozygous gatekeeper mutation FGFR3 V555M appeared as a mechanism of acquired resistance to selective FGFR inhibitors [73]. Several other activating mutations were identified in FGFR2-expressing cells treated with high concentrations of dovitinib, and the multi-kinase inhibitor ponatinib inhibitory activity was affected by the V565I gatekeeper mutation [74]. In addition, a previously undescribed FGFR3 variant was identified as a key contributor to resistance in the MGH156-1A cell line derived from a patient with acquired resistance to EGFR TKIs [75], and follow-up studies clearly indicated that FGFR inhibitors re-sensitized these cells to EGFR inhibitors.

Besides secondary mutations in the kinase domain, resistance to FGFR kinase inhibitors may also occur through activation of alternative signaling pathways, as demonstrated by Harbinski et al. [76] who showed a broad and versatile potential for tyrosine kinase receptor from the FGFR, HER, and MET family to compensate for each other. Javidi-Sharifi et al. [77] suggest that some patients with gastrointestinal stromal tumor (GIST) treated with imatinib can develop a functional dependence on FGFR3, illustrated by the fact that the addition of the FGFR3 ligand FGF2 to GIST cells restored KIT phosphorylation during imatinib treatment. Furthermore, signaling crosstalk between KIT and FGFR3 activated the MAPK pathway to promote resistance to imatinib. *FGFR* amplification and overexpression have also been related to poor prognosis and endocrine resistance in breast cancer [78]. Of note, relationships between cyclins and FGF/FGFRs have also been reported at the protein level. For instance, a study showed that FGFR4 contributed to the maintenance of CCND1 via the mTOR translation pathway, and several other studies demonstrated cooperation between FGFR and CCND1 [79]. Finally, most patients with advanced cancer have complex molecular portfolios, and hence there may be multiple genomic drivers that are active and supplant the role of FGFR [80–82]. In that context, it appears evident that identification of resistance mechanisms is crucial to crafting effective drug combinations.

## Conclusion

The FGF/FGFR pathway is crucial to a variety of human diseases. There are five known FGFRs, FGFR1–FGFR4 and FGFRL1, and 18 human ligands for FGFRs. FGFR germline mutations (activating) can cause skeletal disorders, primarily dwarfism (generally mutations in FGFR3) and craniofacial malformation syndromes (usually mutations in FGFR1 and FGFR2). Loss-of-function mutations in FGF signaling are seen in congenital hypogonadotropic hypogonadism (including the Kallman syndrome variant with anosmia). Interestingly, many of the aberrations that cause the inherited skeletal disorders are also seen in human cancers.

The most common abnormalities in malignancies are gene amplifications of FGFR1–3 or of the FGF ligands. The cancers in which FGFR gene amplifications are most frequent include squamous cell lung cancer (FGFR1), head and neck squamous cell cancer (FGFR1), bladder (transitional cell) cancer (FGFR1), endometrial cancer (FGFR1), gastric adenocarcinoma (FGFR2), breast adenocarcinoma (FGFR1), and prostate adenocarcinoma (FGFR1). Point mutations are seen in all FGFRs but are less frequent in FGFs. For instance, mutations in FGFR3 are frequent in bladder carcinoma, and FGFR2 mutations in endometrial cancer, melanoma, and gastric tumors (Tables 2 and 3).

Interestingly, somatic mutations in FGFR3 have been observed in benign skin conditions such as seborrheic keratosis and epidermal nevi (but not in adjacent normal skin) [54]. FGFR rearrangements are also observed in certain cancers and characterize certain myeloproliferative disorders (Table 2).

Importantly, there are several pharmacologic agents that have been or are being developed for inhibition of FGFR kinases. These include both highly selective inhibitors as well as multi-kinase inhibitors. Ponatinib, regorafenib, pazopanib, and lenvatinib are already FDA-approved for cancer, albeit not on the basis of their FGFR activity. Very few studies in cancer have been aimed at an FGFR biomarker-selected population, and several of the FGFR inhibitors have failed phase III studies in unselected patients. A multi-kinase inhibitor (nintedanib), which suppresses FGFR1–3, was also recently FDA-approved for idiopathic pulmonary fibrosis.

Whether or not FGFR inhibitors could also be used to moderate the phenotype of inherited disorders due to FGFR activation is an intriguing question. Of interest, Garcia et al. [48] injected ia mouse model of achondroplasia with a soluble form of human FGFR3 (acting as a decoy receptor and preventing FGF from binding to mutant FGFR3), and effective maturation of growth plate chondrocytes was restored in the bones of treated mice. Of interest in this regard, individuals afflicted with the inherited disorders associated with FGFR aberrations, such as dwarfism, do not have an increased incidence of cancer, despite having mutations that are often identical to those somatic FGFR aberrations that characterize certain tumors. The secondary modulatory influences that mitigate the risk of cancer in these individuals are of interest. Whether or not treating them at an early age with FGFR inhibitors would increase the later risk of cancer if the inhibitors were withdrawn would need to be considered.

In summary, perturbations in the FGF/FGFR machinery appear to underlie a variety of inherited syndromes, as well as benign and malignant disorders. The advent of potent FGFR inhibitors provides important new agents in the armamentarium against diseases caused by FGF/FGFR abnormalities.
